# A Real-Time Cardiac Arrhythmia Classification System with Wearable Sensor Networks

**DOI:** 10.3390/s120912844

**Published:** 2012-09-21

**Authors:** Sheng Hu, Hongxing Wei, Youdong Chen, Jindong Tan

**Affiliations:** 1 Department of Mechanical, Aerospace and Biomedical Engineering, The University of Tennessee, Knoxville, TN 37996, USA; E-Mails: shengh@mtu.edu (S.H.); weihongxing@buaa.edu.cn (H.W.); tan@utk.edu (J.T.); 2 School of Mechanical Engineering and Automation, Beihang University, Beijing 100191, China

**Keywords:** wearable ECG, cardiac arrhythmia classification, hidden Markov model

## Abstract

Long term continuous monitoring of electrocardiogram (ECG) in a free living environment provides valuable information for prevention on the heart attack and other high risk diseases. This paper presents the design of a real-time wearable ECG monitoring system with associated cardiac arrhythmia classification algorithms. One of the striking advantages is that ECG analog front-end and on-node digital processing are designed to remove most of the noise and bias. In addition, the wearable sensor node is able to monitor the patient's ECG and motion signal in an unobstructive way. To realize the real-time medical analysis, the ECG is digitalized and transmitted to a smart phone via Bluetooth. On the smart phone, the ECG waveform is visualized and a novel layered hidden Markov model is seamlessly integrated to classify multiple cardiac arrhythmias in real time. Experimental results demonstrate that the clean and reliable ECG waveform can be captured in multiple stressed conditions and the real-time classification on cardiac arrhythmia is competent to other workbenches.

## 1. Introduction

Several factors have converged to create new opportunities to address the efficient in-home healthcare system. First, more and more people are suffering the chronic deceases. Cardiovascular diseases (CVDs) have become one of the leading underlying causes of death in both developing and developed countries. A recent report from American Heart Association claims that 81.1 million American adults (more than one third) are estimated having one or more types of CVDs, and they account for 34.3% deaths in 2009 as the underlying causes [1]. Moreover, the health expenditure with established healthcare delivery have been dramatically rising. United States is one of the developed countries that have highest growth rates in the healthcare costs. The share of GDP devoted to healthcare has grown from 9% in 1980 to 16% in 2008 [2]. Last, as the baby-boomers have reached their retiring age [3] in the United States, the long term in-home care are becoming an urgent issue. The aging of population is also an evident trend in other developed and developing countries. Therefore, efficient long term monitoring systems are motivated for personal in-home healthcare.

A traditional monitoring system usually uses a direct-wired connection between the subject and the instrument; the subject is therefore confined to the monitoring instrument. Hence wearable wireless sensors for the monitoring of physiological signals attracts more and more attentions. It detaches the sensing unit from the processing unit, hence the subject with wearable sensors can be monitored in a free living environment. A conventional monitoring system is usually only used for collecting data, while the data processing and diagnosis are done offline [4]. In contrast, the on-body sensor node in the wireless monitoring system can detect early abnormalities by local computational capacity, even when the subject is unaware and unconscious [5]. For the aging people with heart deceases, emergencies often occur when the patients are sleeping. Hence it is important that the medical disorders can be detected early. Finally, using the telecommunication infrastructure [6], signals can be directly delivered to the clinics, where doctors can remotely analyze real-time physiological status of the subject, and make correct diagnosis timely.

There exist technical challenges for the application of wearable wireless sensors in pervasive health-care. First, the clear and stable physiological signal is the paramount concern. Taking ECG as an example, the accuracy of further processing and cardiac arrhythmia classification are all based on the quality of raw ECG morphology. Therefore, analog front-end for physiological signal sensing should carefully designed such that most noises are canceled out before they are fed into an analog-to-digital converter (ADC). Second, most of the existing wireless monitoring systems only consider monitoring single physiological signal [7,8]. Sometimes, multiple physiological statuses need to be monitored simultaneously for accurate diagnosis. For example, when a subject's respiration rate is detected higher than normal, it is possible that the subject is having exercise at that time, or the subject is suffering heart attacks. Third, the wearable ECG sensor node needs to be energy efficient. Energy efficiency considerations include low power hardware and software design, and power efficient networking. All components in this system are carefully chosen with the sleep mode, such that they can be configured into sleep mode according to the requirements of applications. Last, the wearable sensor node needs to have low weight and small size to not to hamper the patient's daily life.

To address these technical challenges, an intelligent Body Sensor network (iBoSen) for biological monitoring and logging is proposed. The subject is monitored by a wearable sensor node in an un-obstructive way that one's daily life will not be interrupted. All physiological signals are digitalized by the on-node ADC and transmitted to a smart phone via Bluetooth. On the smart phone, the waveforms are visualized and corresponding diagnosis approaches are applied to infer the human health status. Once the system detects the patient's abnormalities, the alarm message will be sent to the caregivers or clinics by the embedded Wi-Fi or 3G interface on the phone. The high-level goal of this system is to enable the long-term continuous physiological signal monitoring and real-time detection of multiple cardiac arrhythmias. The contributions of this system are highlighted as follows:

- iBoSen is able to collect the various real-time physiological signals simultaneously: electrocardiogram (ECG), electroencephalogram (EEG), respiration rate and skin temperature. Moreover, an extension socket is for additional signals.
- Energy efficiency considerations of iBoSen include low power hardware and software design, and power efficient networking.
- An improved ECG analog front-end circuit is designed aiming at enhancing the anti-noise ability.
- A novel layered hidden Markov model is proposed to detect cardiac arrhythmia from sensor readings directly. The bottom layer extracts the features of ECG waveform and human activities, and the top layer classifies the type of cardiac arrhythmias simultaneously.
- The physiological signals and human health status are visualized on a smart phone in real time.
- The smart phone could access the remote clinical database using its Wi-Fi or 3G interface.

## 2. Design Overview

**Figure 1.**
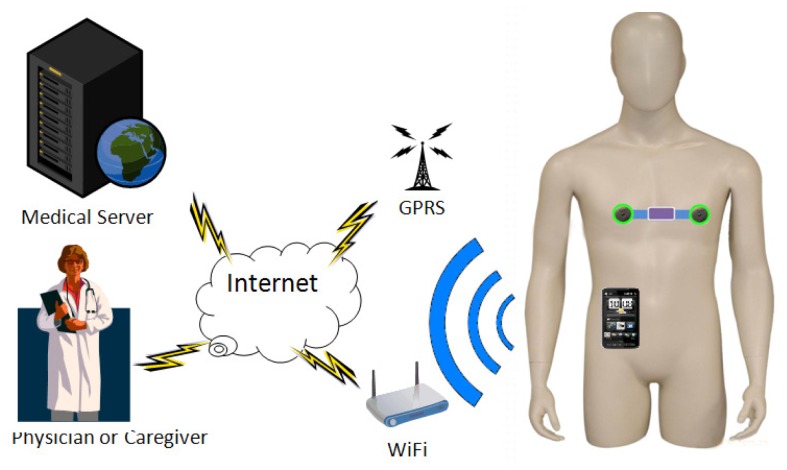
The architecture of iBoSen.

**Figure 2.**
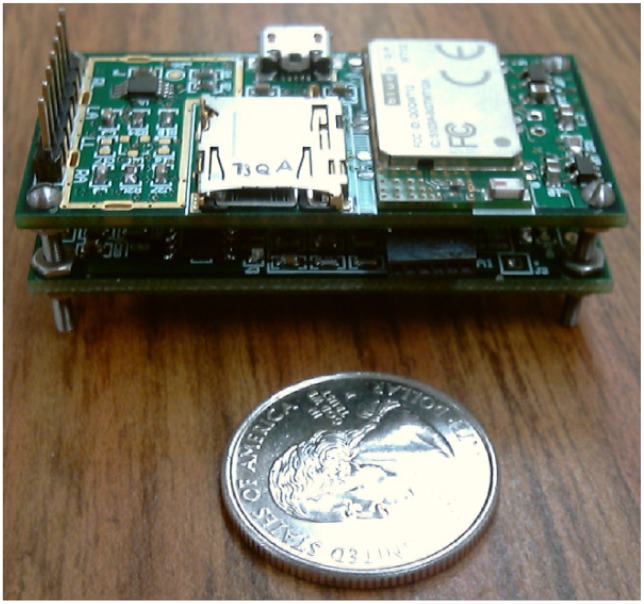
One sensor node in iBoSen.

The iBoSen system consists of two parts: multiple wearable sensor nodes and a smart phone as a Gateway, as shown in Figure 1. Sensor node (Figure 2) attached to the subject's body acquires the physiological signals from the subject, and converts them to digital data. These data can be stored in MicroSD card on sensor node, preprocessed using the on-board computational capability, or transmitted to Gateway using Bluetooth.

The gateway visualizes the physiological signals and is open for various real-time physiological signal based approaches for diagnosis. Once Biologger detects the subject's abnormalities, the alarm message will be sent to the caregivers or clinics by the embedded Wi-Fi or GPRS interface on Gateway

The following of this chapter will be organized as follows. Each module on sensor node will be discussed sequentially, and then we will introduce how Gateway receives the data and how to process these data. After that, a case study of cardiac arrhythmia classification will be presented.

### 2.1. Overview of the Sensor Node Design

**Figure 3.**
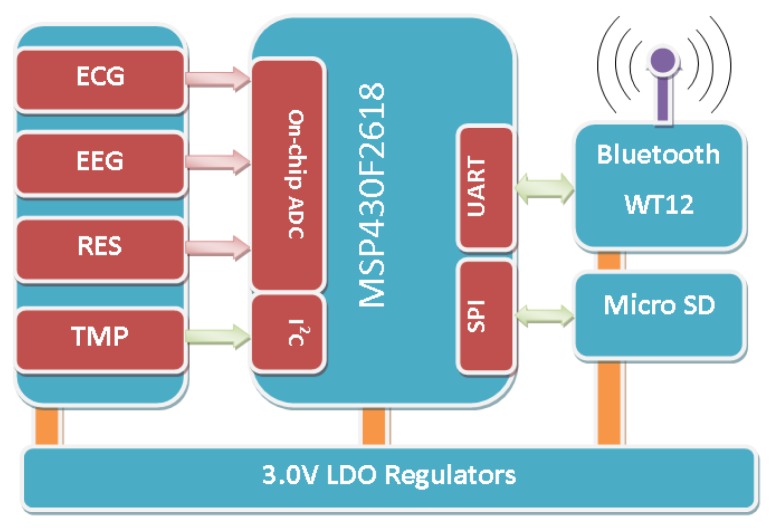
The block diagram of sensor node in iBoSen.

The wearable sensor node consists of seven modules: analog front-end circuits for four physiological signals, a radio communication module, a storage module, and a microcontroller unit (MCU), as shown in Figure 3. The raw signals from the electrodes are fed into corresponding analog front-end circuit for noise decoupling, and then they are amplified to a proper dynamic range and fed into the on-chip analog-to-digital converter (ADC). After that, the MCU can process the data on the specific requirements. The storage module can save all the data on a MicroSD card and the radio module can transmit data wirelessly for monitoring. The specifications are summarized in Table 1.

#### 2.1.1. Microcontroller

MCU plays a critical role in sensor node. Sensor node embeds a 16-bit RISC MCU, MSP430F2618, whose architecture combined with five low-power modes is optimized to achieve extended battery life in portable sensing applications. The device features the digitally controlled oscillator (DCO) which is able to wake up from low-power modes to active mode in less than 1 *μs*. MSP430F2618 has two built-in 16-bit timers, a universal serial communication interface, 10-bit A/D converter with integrated reference and data transfer controller (DTC), two general-purpose operational amplifiers in the MSP430F22x4 devices. MCUs from MSP430 family have been widely applied to various sensor networks (Tmote Sky [9] *etc.*), from which the previous reference design helps save the developing cost.

#### 2.1.2. Wireless Network and Battery

Because the on-body sensor node is attached to subject's body, the main components selected must be small in size and have low power cost in terms of the hardware design. Ultra-low power MSP430F2618 of MSP430 series is a 16-bit RISC (Reduced Instruction Set Computer) processor from TI. Many merits, such as ultra-low power, low cost, rich integrated peripherals, make it widely applied to sensor nodes. There are intensive discussions on networking and communication technologies for body area networks. In this particular design, a low power Buletooth module is used to make it easily accessible with smart phones. The prototype is designed to fit with any polymer rechargeable batteries. With a 500 mA rechargeable battery, a sensor node can be used as a Holter for 24 h. Data is stored locally on the sensor node with occasional transmission between a sensor node and the gateway. The power consumption breakdown is shown in Table 2.

### 2.2. Overview of the Gateway Design

The gateway in iBoSen has three functions. First, it serves as a data sink which is a terminal gathering all the data from sensor nodes. Second, it is capable of analyzing the data from sensor node and making the correct diagnosis. Last, it behaves as an interface to the telecommunication infrastructure via Wi-Fi and 3G network, from which the physiological signal is able to be transferred to the medical care service for further diagnosis or the physician can monitor the subject remotely in real-time. Therefore, three requirements are proposed for the gateway. First, the gateway can support both the short range wireless communication with sensor node and wireless Internet access. Second, it has powerful computational capability to deal with the physiological signal real-time. Third, it must be a portable device such that there is no impact on the subject's daily life. Before the prototype is brought up, we choose the smart phone to serve as Gateway rather than developing a brand new evaluation board. A few factors are concerned during the decision. First, the smart phone on the market meets the requirements mentioned above. Second, using the existing smart phone can reduce the cost of development. Last, carrying a smart phone has been a part of human's daily life, other forms of gateways will be an extra burden for the subject.

Among the amount of the off-the-shelf smart phones, an HTC HD2 with a 1 GHz CPU and 448 MB RAM is chosen, although any types of smart phones such as an iPhone or an Android phone could act as the gateway. Window mobile operating system offers almost the same API with the ones on the desktop version, therefore we can transplants the desktop software to the mobile device easily. In addition, as Visual Studio has already supported the embedded system development, we can develop the user interface on the smart phone seamlessly. Last, the development on the Windows mobile is free and the developer does not need to pay for the authority.

#### 2.2.1. Realtime Physiological Signal Display

**Figure 4.**
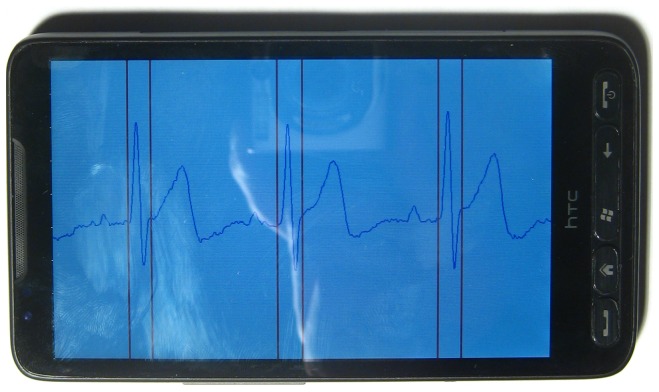
HTC HD2 collecting data from sensor node.

As real-time digitalized data arrives at the phone, the Bluetooth communication thread relays the data to the display thread for plotting on the screen. Multiple threads are employed to display multiple sensor nodes. Another thread manages a responsive GUI and increase thread-level parallelism. The ECG signal is plotted on screen as shown in Figure 4. The waveform can be zoomed in/out by sliding the screen such that the user is allowed for a close-up examination of the physiological signal. The scale of the axis for the ECG plotting follows the clinical standard of a resting ECG machine: the scale for the vertical voltage axis is 0.5 mV per division, and the scale of the horizontal time axis is 200 ms per division. Meanwhile, each beat is classified and annotated by two read line showing each QRS complex.

#### 2.2.2. Realtime Diagnosis Platform

The framework of the user interface only provides the basic functionality to communication setup and waveform visualization. More realtime diagnosis approaches can be embedded into this framework as an plugin. These approaches could be implemented as a separate dynamic linked library (dll) from the main application and runs on its own thread. Using this framework, multiple dll plugins can be created for different vital sign analysis and realtime diagnosis without changing the main program, which is responsible for data acquisition and display.

## 3. Analog Front-end Design

For any mix-signal embedded system, ensuring proper connections to the analog signal of interest and ground are essential to obtaining accurate measurements. Therefore, the analog front-end, as the direct touch of the raw signal, is the first concern in a system design. If the analog front-end is not well designed, the reconfigurability of the entire system is at stake. In this section, we will focus on the ECG and EEG analog front-end circuits.

### 3.1. Noise Handling and Grounding

For an ECG monitoring and diagnosis system, any noise on ECG waveform will limit the resolution of further analysis and diagnosis. Hence, the ECG analog front-end circuit design is the paramount concern in most ECG systems. The unconditioned ECG from the electrode is an extremely weak signal ranging from 0.5 mV to 5.0 mV, while the magnitude of the coupled noises could be up to ±300 mV. Therefore, the noise must be handled in order to pick up the valid ECG waveform. Most noise of wearable ECG are associated with the following resources:

- The large common mode noise results from the potential between the electrodes and ground.
- The great DC offset resulting from the electrode-skin contact limits the performance of the instrumentation amplifier.
- The system can pick up the electrical noise (50 Hz in America and 60 Hz in most parts of the world) at any point along with the signal flow.
- The changes of the impedance and capacitance are sensed by the ECG electrode and result in motion artifacts.
- The radio frequency interference (RFI) can be coupled into ECG along the leads.

**Figure 5.**
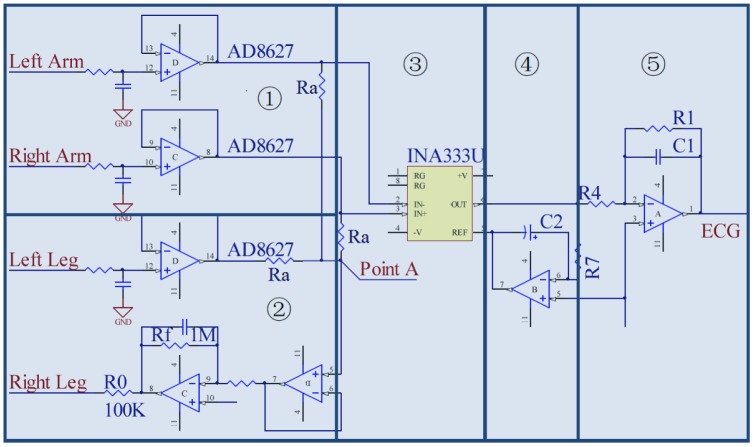
The schematic design of the ECG analog front-end circuit. ➀ Two JFET input voltage followers; ➁ The driven right leg circuit; ➂ Instrumental amplifier, INA333; ➃ An AC coupled circuit; ➄ An active filter.

To deal with these noises, the ECG analog front-end design in this sensor node gives the complete solution, as shown in Figure 5. A voltage follower in Figure 5(1) is plugged in the each input channel. This is due to the fact that the input bias current (*I_b_*) of the front-end amplifiers can polarize the electrode if there is poor skin contact: Δ*v_e_* = *I_b_*·Δ*z_e_*, where Δ*v_e_* is variation of the voltage at the electrode site and Δ*z_e_* is the equivalent impedance variation. Therefore, AD8627, a JFET input operational amplifier with input bias currents less than 1 pA is selected here to reduce the influence by the impedance variation at the electrode site. The RC network before AD8627 is used for electro-static discharge (ESD) protection. By plugging in these two voltage followers, the interference of motion artifact will reduce a lot. Figure 5➀ depicts degradation of the ECG waveforms under different stress conditions (these waveforms are not processed by the digital filter on node). From this figure, it can be concluded that the key features can be still identified with slight activities. However, when the patient is jogging, only QRS complex in the ECG waveform can be detected reliably.

Moreover, most ECG front-ends are 3-lead configuration circuits, in which the third lead is a driven right leg circuit for injecting the inverted common mode noise back into the body to cancel them out. Unfortunately, the reason why the driven right leg circuit is required and how it works are not well explored in many designs, which results in non-optimality in these designs. Actually, the driven right leg circuit can provide a low impedance path between the patient and the amplifier, such that less common mode noise will be transformed by the amplifier. Through the calculation in [11], if *G* is defined as the resistor ratio of the inverting amplifier, $G=\frac{2\cdot R_{f}}{R_{a}}$ in the traditional driven right leg circuit (Figure 1 in [11]). *G* needs to be set as large as possible to minimize the common mode voltage *v_c_*.

**Figure 7.**
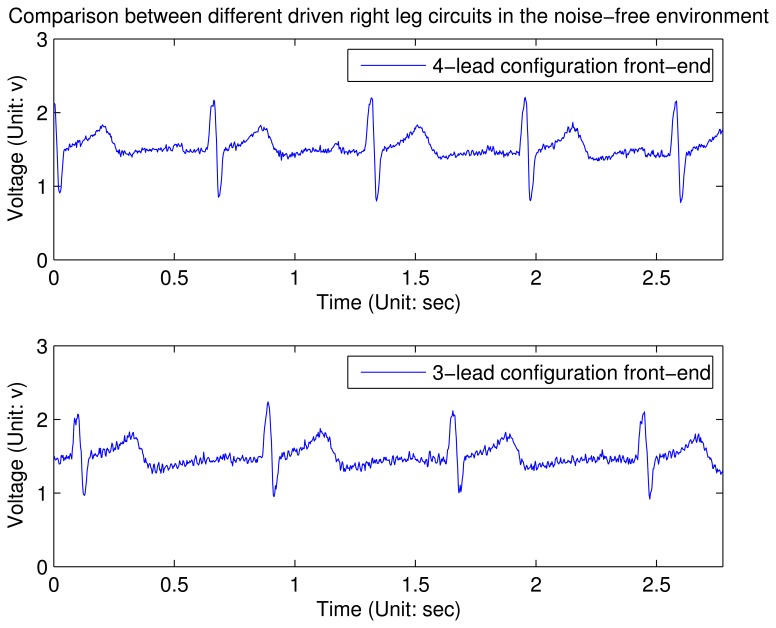
ECG waveforms using different driven right leg circuits in the noise-free environment. The charts show a three-second snapshot.

**Figure 8.**
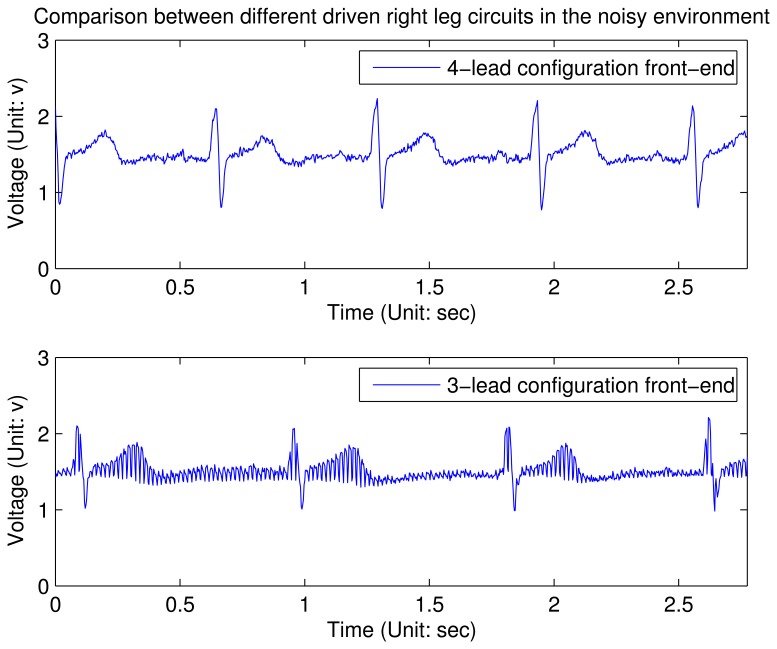
ECG waveforms using different driven right leg circuits in the noisy environment. The charts show a three-second snapshot.

In contrast to the typical driven right leg circuit, a novel driven right leg circuit is proposed, as shown in Figure 5➁. We name the circuit 4-lead configuration front-end, where the fourth lead is connected to the left leg of the body. The signal along this channel will not be amplified but only provides an bias at point A. Therefore, *G* is modified to $G\prime =\frac{3\cdot R_{f}}{R_{a}}$ due to the third input channel, such that *G′* is 1.5 time to *G* when other parameters do not change. Empirically, we find that 100 is a proper value for the gain, since a higher value will affect the stability of the system. Therefore, *R*_0_ is set to 10 kΩ and *R_f_* is set to 1 MΩ in this design. From the experimental results shown in Figures 7 and 8 (these waveforms are not processed by the digital filter on node), it can be concluded that in an clear and noise-free environment, the performance of the modified driven right leg circuit is the similar to the traditional one [11]. However, when the wearable sensor node is placed in a high interference environment, the proposed circuit largely outperforms the traditional one.

Other methods for suppressing the noise and removing DC offset include:

- One INA333 with a high common mode rejection ratio (100 dB) is placed in the first stage of amplification, as shown in Figure 5➂. Its structure with three operational amplifiers makes it reject the common mode noise. In addition, it contains an internal RFI filter to get rid of most RFI.
- An AC coupled circuit in Figure 5➃ is designed to remove the large DC offset, such that the gain of operational amplifier in Figure 5➄ can be set as large as possible. According to our experience, setting the cutoff frequency, $f_{c}=\frac{1}{2\pi C_{2}R_{7}}$, to be 0.5 Hz has the best performance. If the frequency were lower than 0.5 Hz, the capacitor would be charged for a long time, resulting in the operational amplifier saturated.
- An active filter is shown in Figure 5➄. Its cut-off frequency is set to be 0.5–150 Hz [12] and the gain is set to be 100, such that the dynamic range of conditioned ECG waveform would be 2 V, which is two third of the rail-to-tail voltage of the amplifier.
- The proper layout grounding can reduce the level of 50/60 Hz noise, especially in a severe environment.

### 3.2. ECG On-Node Filtering

Although many approaches are explored in the analog front-end circuit to remove the 50/60 Hz noise, the noise can be coupled into analog signal flow at any point. Therefore, a digital filter is a better solution than the filer in analog domain. Due to the limit of computational capability, one second order IIR with a notch frequency at 50/60 Hz and a 3 dB notch bandwidth of 6 Hz [13] is implemented on the node. Taking the 50 Hz filter as an example and assuming a sampling frequency of 360 Hz, the normalized angular notch frequency and the normalized angular 3 dB bandwidth can be calculated as

(1) $$\omega_{0}=2\pi (\frac{50}{360})=\frac{5\pi }{18},\omega_{0}=2\pi (\frac{6}{360})=\frac{\pi }{30}$$

Therefore, the desired transfer function can be obtained by

(2) $$H(z)=\frac{1-1.287z^{-1}+z^{-2}}{1-1.223z^{-1}+0.900z^{-2}}$$

**Figure 9.**
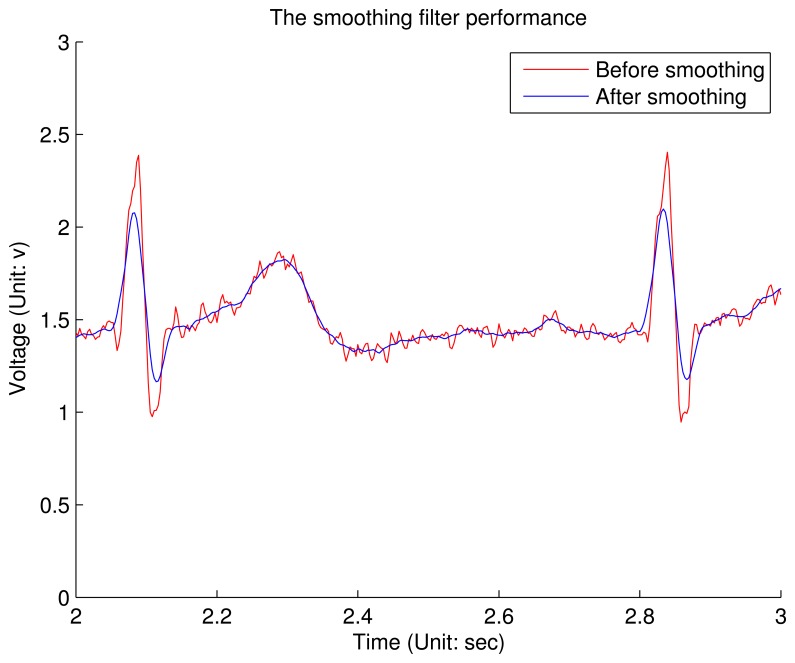
The performance of the SG filter. The chart shows a one-second snapshot.

However, the output of this notch filter contains some small ripples along the signal, which is called the Gibbs phenomenon. It shows that the amplitude of these ripples can not be reduced by increasing the order of the filter. Therefore, a Savitzky–Golay smoothing filter (SG filter) [14] is integrated on the node. The main advantage of SG filter is that it tends to preserve features of ECG, such that P subwave will not be lost. According to our experience, the SG filter with the zero-order polynomial regression and 15-point sliding window is optimal when the trade-off between ripple amplitude and subwave preservation is considered. Figure 9 shows the comparison between the SG niter's input and output. It can be conclude that although the amplitude of QRS complex is shrunk, most ripples are removed and all the key features are preserved.

## 4. Firmware

**Figure 10.**
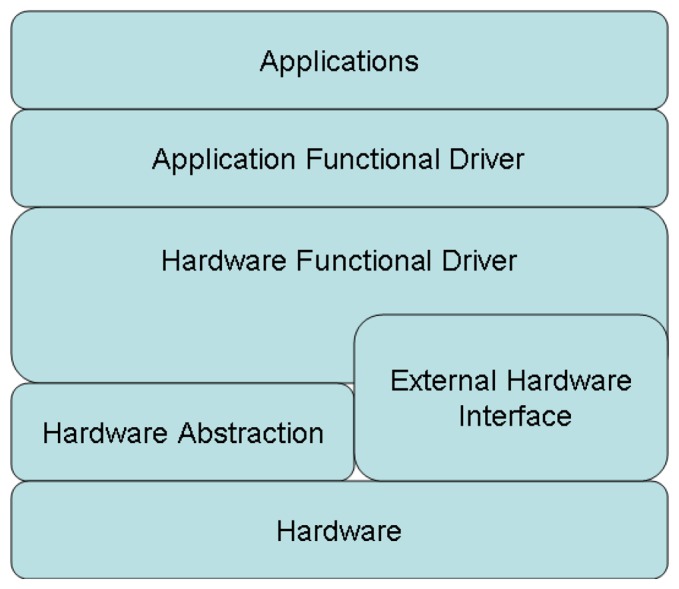
The architecture of the firmware on sensor node.

Firmware is the program running on the MCU, which interacts with all other modules on the MCU. According to the general embedded system developing principle, the hierarchical architecture is applied to the firmware on sensor node as shown in Figure 10. The firmware is hierarchized to multiple layers per their functionality. This is a multi-layer structure, every layer fulfills their own functionalities, and the communication between two layers are based on the interface. Basically, the applications or functions only rely on the layers below it. This makes most codes independent of the hardware and helps to implant across different hardware platforms.

### 4.1. Hardware Layer

The hardware layer is lying on the bottom of the system. It contains both the on-chip functional modules and peripheral sensors.

### 4.2. Hardware Abstraction Layer

The hardware abstraction layer (HAL) is an abstraction layer, implemented in software, between the hardware layer of the MCU and the driver layer that runs on that hardware platform. Its function is to hide differences in hardware from most functional driver, so that most of the driver code does not need to be changed to run on systems with different platforms. On a PC, HAL can basically be considered to be the driver for the motherboard and allows instructions from higher level computer languages to communicate with lower level components, such as directly with hardware. In sensor node, the HAL is much simpler: it generally contains register write/read operations and interruption handling.

### 4.3. Hardware Functional Driver Layer

This layer envelops the basic on-chip module primitive operations and provides the interface to the upper layer. Take the SPI module in the MSP430 as an example. The hardware functional driver layer contains functions like SPIStart(), SPIWriteByte(), SPIReadByte() and SPIStop(). Meanwhile, this level also contains the external sensor interface, which provides the way to access the peripheral sensor on the board.

### 4.4. Application Functional Driver Layer

This layer offers the APIs for the applications on the top level. In this level, all the hardware related access are mapped into the memory space. The MCU accesses it as the memory access. The applications can call those functions in this layer easily. For example, the accelerometer operations and data exchange, like InitialACC() and getACCData() and stopACC(), are placed in this layer.

### 4.5. Application Layer

A simple real-time task scheduler is implemented on sensor node to manage three periodic tasks: (1) acquiring physiological signals; (2) preprocessing the digitalized signals and (3) encoding and transmitting the data to base station. In addition, if some abnormalities are detected in the second task, a non-exemptible sporadic task with a higher priority is released to sound alarm. The abnormalities can be defined by the users in advance.

## 5. Cardiac Arrhythmia Classification with iBoSen

**Figure 11.**
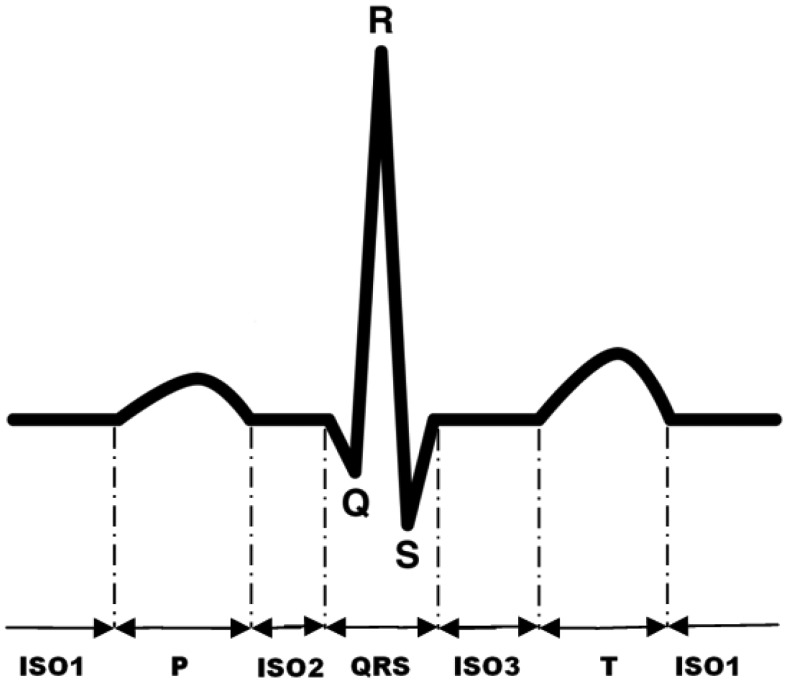
ECG waveform and its subwaves (P subwave, QRS complex, T subwave and isoelectrics) in a cardiac cycle.

This is a case study in which we develop a novel real-time cardiac arrhythmia classification approach on Gateway. Gateway receives the clear and reliable ECG for visualization and cardiac arrhythmia detection. Among the numerous cardiac arrhythmia classification paradigms, we focus on the hidden Markov model (HMM) based algorithms. First, a typical ECG period (Figure 11) always consists of a P subwave, a QRS complex, a T subwave and isoelectrics between those subwaves. HMM is also able to preserve the ECG structural nature. Second, the HMM is more adaptive to the dynamics than the heuristic rules. The HMM belongs to a probabilistic module, hence an absolute threshold is not required. Third, the HMM has relatively low cost in time complexity, which is more favored in an embedded system. Finally, the HMM can be used in the non-linear system, while the extra errors are introduced when using KF methods [15].

However, two issues are not concerned in the existing HMM-based algorithms [16–18]. One is the interference from motion artifacts. Motion artifacts also can distort the morphology of ECG waveform, thus false alarm will be triggered if they are not treated properly. In our design, an accelerometer is introduced in the algorithm to classify the human motion, such that the ECG waveform distorted by the artifacts cannot be identified as an arrhythmia. Another problem is the big time delay. Generally, the process of cardiac arrhythmia detection contains two steps: the first step is ECG segmentation or ECG feature extraction, the second one is to classify the type of arrhythmias based on the results inferred from the first step. In this way, the response time would be increased, and the abnormalities could not be found timely. To deal with this problem, a novel layered hidden Markov model (LHMM) framework is proposed in our system to real-time identify multiple cardiac arrhythmias.

**Figure 12.**
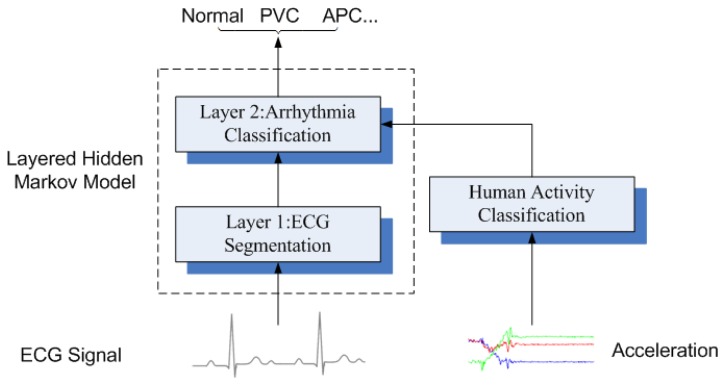
The block diagram of the proposed LHMM framework.

The LHMM is derived from the normal HMM, where the HMM on upper layer corresponds to observation symbols or probability generators at the lower layer and in the bottom layer, primitive observation symbols will be yielded directly from observations of the modeled process. The proposed model consists of two layers of HMMs. The ECG waveform is fed into Layer 1 HMM and used to segment the ECG waveform. Meanwhile, Layer 2 HMM is responsible for detecting the types of cardiac arrhythmias using the ECG clinical features and information from the accelerometer as observations, as shown in Figure 12.

### 5.1. Layer 1: ECG Segmentation

ECG segmentation is the first step to diagnose the cardiac arrhythmias. The onsets and offsets of characteristic subwaves need to be localized precisely from the periodic ECG signals. Layer 1 HMM is used to fulfill this task and extract clinical ECG features.

#### 5.1.1. Model Description

**Figure 13.**
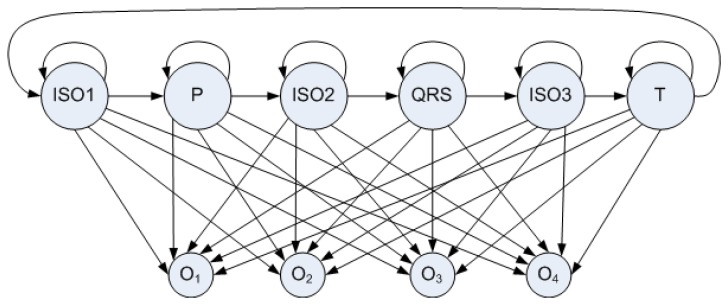
State space and observation space in Layer 1 HMM. “P” stands for P subwave, “QRS” for QRS complex, and “T” for T subwave. Readings from 0 to 1,023 correspond to *O*_1_, readings from 1,024 to 2,047 corresponds to *O*_2_.

A typical period of ECG waveform can be partitioned into six characteristic subwaves, which are Isoelectricl (ISO1), P subwave, ISO2, QRS complex, ISO3, T subwave, as shown in Figure 11. Therefore, the mapping of ECG waveform to hidden states in the HMM can be done by associating each sample (ADC reading) with a hidden state representing a characteristic subwave. Figure 13 shows the six-element state space defined in Layer 1 HMM. As to the observation space in Layer 1 HMM, the 12-bit ADC readings can not be fed into HMM directly, since the value the observation space ranging from 0 to 4,095 is too large for a real-time HMM algorithm. Hence ECG signal is classified into 4 levels according to the magnitude of the corresponding voltage value. Each level associates with a distinct observation symbol (*O*_1_, *O*_2_, *O*_3_, *O*_4_) respectively, as shown in Figure 13.

**Figure 14.**
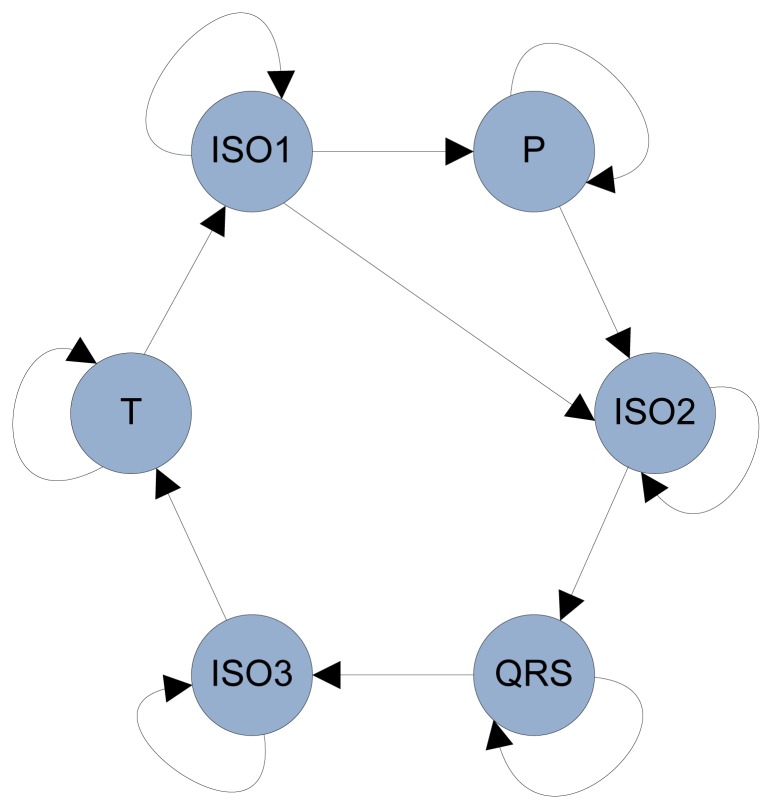
The revised Layer 1 HMM. A connection is added between ISO1 and ISO2.

The model shown in Figure 13 can represent the normal cardiac sinus beats well, but does not take into account the cardiac arrhythmias. On one hand, there exists a problem called double-beat segmentation, in which two or more heat beats are detected incorrectly, when there is only one beat present. To address this problem, a duration constraint is incorporated into Layer 1 HMM. Every state has an empirical minimum duration, such that the HMM cannot generate false heart beat in the constraint range. In our experiment, the minimum state duration is calculated as 60% of the minimum duration in the training data. On the other hand, sometimes P wave would be missing, when cardiac arrhythmia happens. In this case, transition between ISO1 and ISO2 could be possible in terms of the HMM chain. Accordingly, Figure 14 shows the modified HMM chain to adapt more situations.

#### 5.1.2. Automatic ECG Segmentation

**Figure 15.**
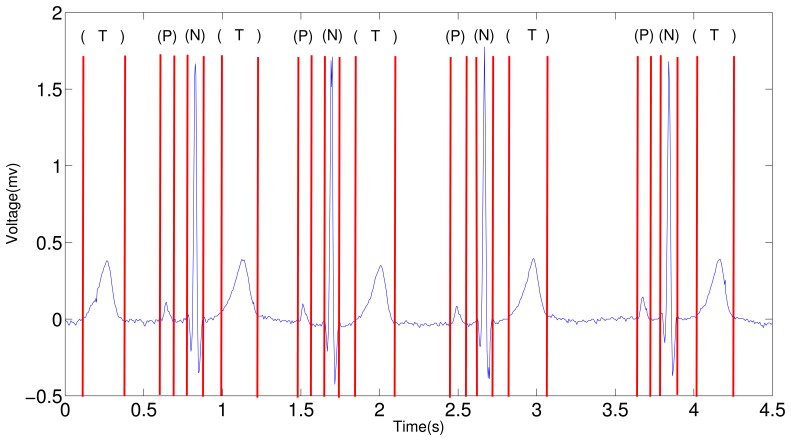
ECG segmentation using Layer 1 HMM. “P” stands for P subwave, “N” for a normal QRS complex, and “T” for T subwave.

The results deducted from Layer 1 in the entire framework is backed up, and Figure 15 shows the four-beat normal ECG waveform and its subwaves (P, QRS, T) in every period. In this figure, the onset and offset of all the subwaves are identified by red lines, the letter on the top indicates the type of the subwave.

### 5.2. Layer 2: Cardiac Arrhythmia Classification

#### 5.2.1. State Space

There are a variety of cardiac arrhythmias, such as Premature Ventricular Contraction (PVC), Atrial Premature Contraction (APC), Right Bundle Branch Block (RBBB) and Left Bundle Branch Block (LBBB). In Layer 2 HMM, two common cardiac arrhythmias are considered: PVC and APC. In contrast to the normal sinus heart beat, they have different characteristics in terms of the ECG morphology.

- PVC is characterized by premature and bizarrely shaped QRS complexes usually wider than 120 ms in width on the ECG. These complexes are not preceded by a P wave, and the T wave is usually large, and its direction is opposite to the major deflection of the QRS.
- APC has an abnormal P wave. Normally P subwave are smaller and rather shapeless. In addition, P subwave occurs earlier than expected, which means P-Q interval is larger than the normal ones.

**Figure 16.**
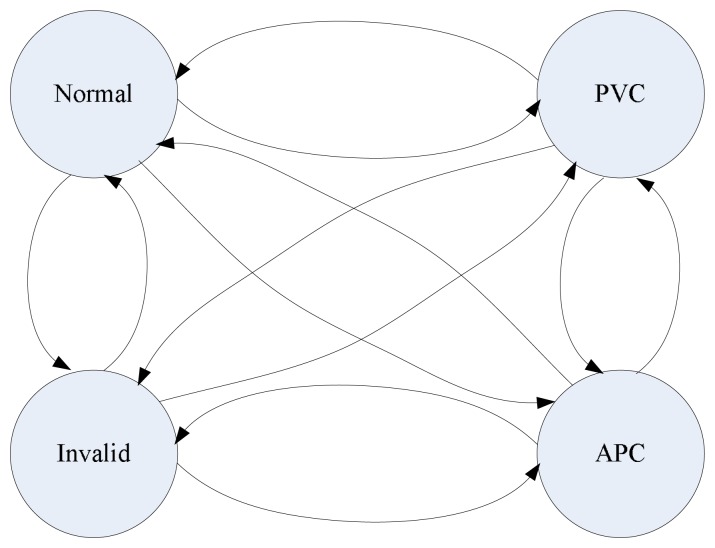
The structure of Layer 2 HMM for cardiac arrhythmia detection. “Normal” means a normal beat,g and “Invalid” means an invalid beat.

Generally, these two types of arrhythmias could occur intermittently. Hence, a fully-connected ergodic model is constructed, as shown in Figure 16. In this model, besides the two cardiac arrhythmias, two other hidden states exist: one is the normal sinus beat, and the other one is invalid beat. Invalid beat means the system has detected strong motion artifacts. The ECG waveform acquired from the front-end circuit can not represent the real heart status.

#### 5.2.2. Observation Space

The Observation Space of Layer 2 HMM contains two parts, as shown in Figure 12. By definition, the hidden state sequence of Layer 1 HMM should serve the observation of Layer 2 HMM. However, PVC and APC classifications do not require the onset and offset of all the subwaves. Therefore, valuable clinical features are extracted from ECG segmentations, such as QRS duration, P-R interval and R-R interval. Sometimes, P wave is skipped and R-R interval will make more contributions to arrhythmia classification.

Another part of observations comes from the tri-axis accelerometer. The accelerometer's Z axis is perpendicular to the earth and points to the earth. Three common activities are concerned during human's daily life: standing, walking and lying, and consider the human activity as an element in Layer 2 HMM's observation space. A simple heuristic-rule algorithm is employed to classify these three activities (Algorithm 1), in which three features are calculated from the accelerometer readings, the acceleration value of Z axis, the sum of squares of accelerations along three axes, and acceleration variance along three axes. The sliding window is about three seconds, which may contain about three to four cardiac periods. Algorithm 1 describes the operation of this algorithm. If the patient is still or moving slowly, the sum of tri-axis acceleration square should be one *g* (gravitational constant). If it goes beyond this value, and the variance is also greater than a particular threshold, the patient is defined to be walking, and ECG segmentation is going to be considered moving. Otherwise, the patient is classified to be standing, when Z axis value is close to one G, and be lying, when its value is close to zero. Under the later two situations, the ECG waveform is considered reliable. Different QRS detection algorithms under varying motion status are used for robust ECG analysis [19].

**Figure 17.**
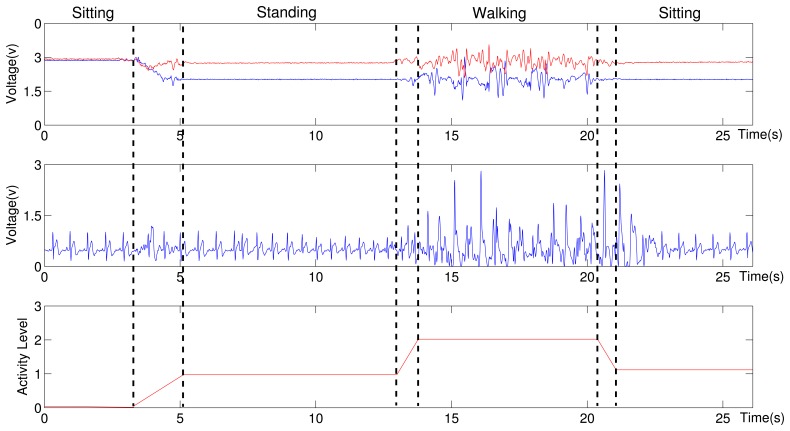
Human activity classification based on the accelerometer measurements. The dashed lines represent the human activities and the transitions between them.

Figure 17 shows the relationship between ECG and acceleration: the upper chart shows a snapshot of the subject's ECG waveform; the middle chart displays the corresponding accelerometer measurements; the lower chart gives the classification result of Algorithm 1. It can be summarized that the subject experiences four stages during the period of more than 25 s. Moreover, the ECG waveform is corrupted during the walking stage.



**Algorithm 1** Human Activity Classification Algorithm.
 **while true do**  Acquire accelerometer readings, *A_x_ A_y_ A_z_* are three components along three axes;  Calculate acceleration 
$A=\sqrt{A_{x}^{2}+A_{y}^{2}+A_{z}^{2}};$;  Calculate *σ*, covariance of *A* in a 3 s sliding window;  **if**
*A* ≥ *δ*_1_
**and**
*σ* ≥ *δ_2_*
**then**   {*δ*_1_ and *δ_2_* are two thresholds.}   **return** Walking;  **else**   **if** |*A_z_*| ≤ *ε*
**then**    {*ε* is a small number closed to zero.}    **return** Lying;   **else**    **return** Standing;   **end if**   **end if** **end while**

### 5.3. Experiments and Performance Evaluation

#### 5.3.1. Experimental Results

**Figure 18.**
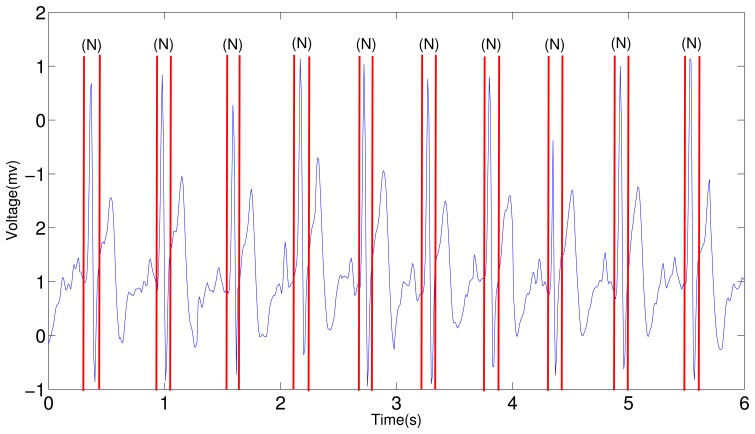
ECG analysis for the wearable ECG sensor node. All the normal beats are annotated as “N”.

The proposed algorithm is implemented on the smart phone using Visual Studio 2008. Figure 18 demonstrates the effectiveness of the system: the ECG waveform is captured by the wearable ECG sensor node. No PVC or APC is present in the waveform during this six second snapshot.

**Figure 19.**
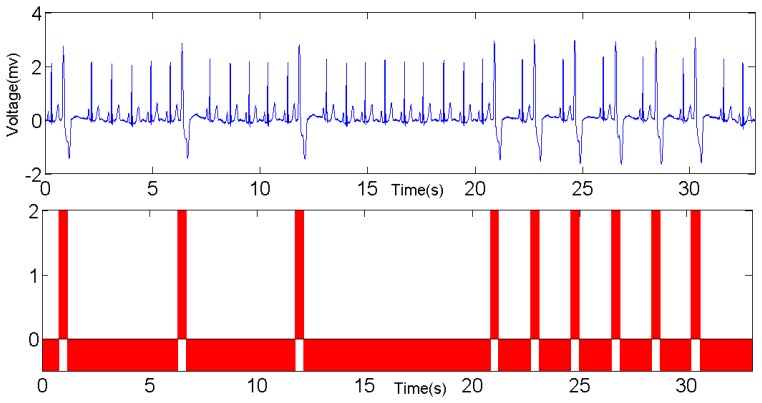
PVC detection based on the proposed LHMM.

**Figure 20.**
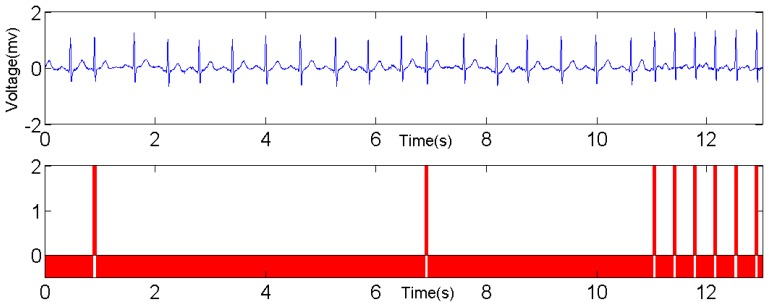
APC detection based on the proposed LHMM.

To compare the reliability and accuracy with other benchmarks, an ECG emulator is also developed on the laptop. It behaves like a wearable sensor node, except that the data are from MIT-BIH Arrhythmia Database [20,21] which is loaded in advance. The ECG emulator also employs the embedded Bluetooth module on the laptop to transmit the data using both the same data format and sending rate with the wearable ECG sensor node. Figure 19 illustrates a snapshot of ECG in Record 119 in [21] and the classification result. In this record, there are 1,987 beats in total, 1,543 normal beats and 444 PVCs respectively. The red bars in the lower chart identify the PVCs in the ECG waveform, which are wider than normal beat. Similar to PVC detection, Record 220 in [21] is used for APC detection and the result is shown in Figure 20.

#### 5.3.2. Evaluation Metrics

The performance of arrhythmia classification is quantified using the widely accepted statistical metrics: accuracy, sensitivity and positive predictivity. Accuracy (Ac), as the most crucial metric, is used to evaluate the overall performance for all kinds of beats. This overall parameter is defined as

(3) $$Ac=\frac{N_{t}-N_{e}}{N_{t}}\times 100\%$$

where *N_t_* represents the beat number in the record, and *N_e_* is the the total number of classification errors. To express the ability of detecting the fraction of events and true events correctly, the sensitivity (Se) and positive predictivity (+P) are defined as follows:

(4) $$Se=\frac{TP}{TP+FN}\times 100\%$$

(5) $$+P=\frac{TP}{TP+FP}\times 100\%$$

where *TP*, *FN* and *FP* denote the number of true positives, false negatives and false positives respectively. True positive means the detection is corresponding to the annotations labeled by experts. False negatives are beats that should be detected but are missed and assigned to another state. A false positive happens when the beat is assigned incorrectly.

To evaluate the overall performance for all records, the weighted measure [22] is used according to the number of beats of each state that exist in the record. It is defined as

(6) $$M^{WA}=\frac{\sum_{i=1}^{n_{f}}n_{b_{i}}*M_{i}^{C}}{\sum_{i=1}^{n_{f}}nb_{i}}\times 100\%$$

where *M* denotes the sensitivity or positive predictivity, *n_bi_* is the number of beats for the *i*th record, *n_f_* is the total number of records, and $M_{i}^{C}$ is the value of a specific measure for the *i*th record in the class “C”, which might be one of Normal, PVC and APC.

#### 5.3.3. Performance

For overall performance evaluation, 16 ECG records are selected from the Arrhythmia group in [21]. These records are all acquired from the modified Lead II. For ease of presentation, these records are divided into 4 types: records with only normal beats (type I), records with normal beats and PVCs (type II), records with normal beats and APCs (type III) and records consisting normal beats, PVCs and APCs (type IV). Three common statistical metrics, *i.e.*, accuracy, sensitivity and positive predictivity, are adopted to quantified the algorithm performance.

Table 3 shows the comprehensive results for PVC and APC detection. For type I, the normal beats are detected accurately. It shows the proposed algorithm is competent for arrhythmia classification. The evaluation results of type II demonstrates that the PVCs are abstracted from the normal beats. The accuracy, sensitivity and positive predictivity even achieve 100% for the records 119 and 230 using the proposed algorithm. Therefore, the algorithm is fully suitable for PVC detection. Similarly, during the APC detection, shown in type III, the algorithm works with the sensitivity of 100%. The performance is better than PVC detection, because morphology of ECG changes a little when APC occurs, while the change of ECG waveform will be significant when there are continuous double PVCs existing especially. The last type is a mixture of PVCs and APCs. In most cases, the sensitivity and positive predictivity are equal to 100%. The overall performance shows the capability of the arrhythmia detection with a weighted average *Se* and +P of 99.72% and 99.64% respectively.

Further, our algorithm is compared with other benchmarks proposed in the literature, as shown in Table 4. Due to the fact that most of them is used for PVC detection, the lateral comparison only concerns PVC detection. It can be seen that the proposed method yields a higher sensitivity, while the positive predictivity of 96.63% is achieved in the acceptable range.

## 6. Summary

This paper presented iBoSen, a wireless physiological monitoring and logging system. It can simultaneously monitor and log various types of physiological signals including ECG. We focus on improving the ECG signal quality by hardware design and propose a real-time cardiac arrhythmia classification algorithm, which successfully monitors the patient's ECG and identifies its cardiac arrhythmia in real time. The ECG waveform and the cardiac arrhythmia classification result are displayed in a smart phone, rather than a bulky resting ECG instrument. The plug-in algorithm can detect the normal sinus beat, PVC and APC at the same time. Once the abnormalities are detected, the alarm message will notify the patient or clinicians via telecommunication infrastructure. Further, the patient can utilize this system to monitor their heart status when the daily lifestyle changes, in such a way that the patient can adjust to living in a comfortable lifestyle and potential risk would be decreased.

## Figures and Tables

**Figure 6.**
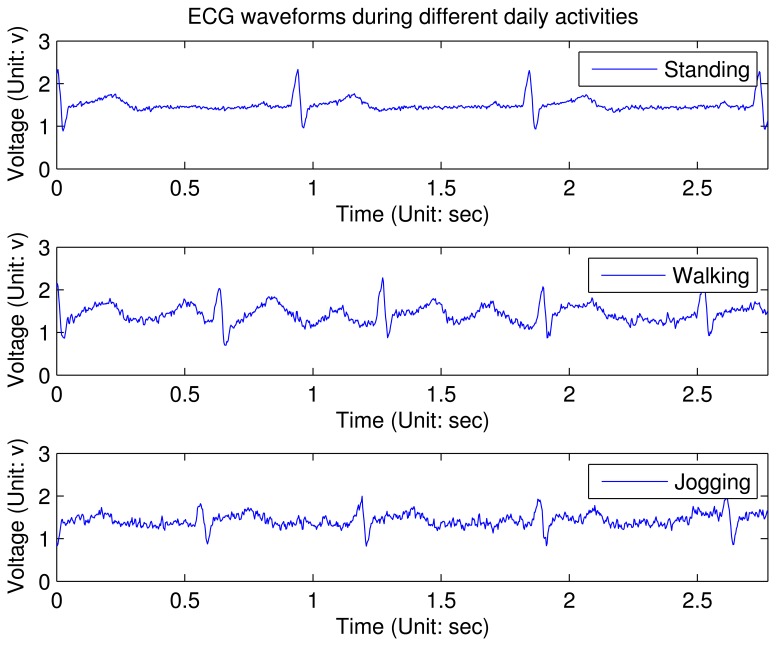
ECG waveforms captured during different daily activities. The charts show a three-second snapshot.

**Table 1.** Specifications of the sensor node.

**Module**	**Parameter**	**Specification**
ECG	Gain	120
	Sampling rate	100 Hz
	Low pass cutoff frequency	40 Hz
	High pass cutoff frequency	0.1 Hz
EEG	Gain	7,680
	Sampling rate	100 Hz
	Low pass cutoff frequency	30 Hz
	High pass cutoff frequency	0.1 Hz
Temperature	Sampling rate	1 Hz
	Sensing Range	−80 to + 150 °C
Respiration	Sampling rate	10 Hz
On-chip ADC	Resolution	10 bit
Radio	Data rate	250 Kbps
Storage	Capacity	2 GB
Power	Voltage	3.0 V

**Table 2.** Power consumption of the parts in iBoSen.

**Parts**	**Operating Status**	**Typical Current**
MSP430F2618	Active Mode@ 1MHz	390 *μ*A
	LPM3@lMHz	0.5 *μ*A
RN 42 [10]	Standby/Idle	25 mA
	Connected (normal mode)	30 mA
	Connected (low power Sniff)	8 mA
	Standby/Idle (Deep sleep enabled)	26 uA

**Table 3.** Results for cardiac arrhythmia classification using the LHMM. *N_b_* denotes the number of beats.

**Type**	**Record**	***N****_t_*	**Ac(%)**	**Normal**			**PVC**			**APC**		

				***N****_b_*	**Se(%)**	**+P(%)**	***N****_b_*	**Se(%)**	**+P(%)**	***N****_b_*	**Se(%)**	**+P(%)**
I	115	1,952	100.00	1,952	100.00	100.00	0	N/A	N/A	0	N/A	N/A
	122	2,474	100.00	2,474	100.00	100.00	0	N/A	N/A	0	N/A	N/A
	106	2,027	93.73	1,507	99.54	96.21	520	98.07	96.15	0	N/A	N/A
	119	1,987	100.00	1,543	100.00	100.00	444	100.00	100.00	0	N/A	N/A
II	123	1,517	99.01	1,514	100.00	99.08	3	100.00	75.00	0	N/A	N/A
	221	2,427	99.56	2,031	99.46	99.93	396	99.24	95.96	0	N/A	N/A
	230	2,255	99.65	2,254	100.00	99.65	1	100.00	100.00	0	N/A	N/A
	101	1,864	99.30	1,859	100.00	99.31	0	N/A	N/A	5	100.00	100.00
	103	2,083	99.95	2,081	100.00	100.00	0	N/A	N/A	2	100.00	66.67
III	112	2,537	99.40	2,535	100.00	99.41	0	N/A	N/A	2	100.00	25.00
	117	1,534	100.00	1,533	100.00	100.00	0	N/A	N/A	1	100.00	100.00
	220	2,046	100.00	1,952	100.00	100.00	0	N/A	N/A	94	100.00	100.00
	100	2,271	99.87	2,237	100.00	100.00	1	100.00	100.00	33	100.00	89.63
	116	2,411	99.34	2,301	99.52	100.00	109	91.74	86.96	1	100.00	50.00
IV	215	3,361	98.51	3,194	98.62	100.00	164	93.29	90.00	3	100.00	100.00
	228	2,053	99.10	1,688	98.93	99.52	362	96.69	100.00	3	75.00	100.00
Total		34,799	N/A	32,655	N/A	N/A	2,000	N/A	N/A	144	N/A	N/A
Weighted Average		N/A	99.20	N/A	99.72	99.64	N/A	97.75	96.63	N/A	99.48	95.77

**Table 4.** Performance comparison on PVC detection. *N* is denoted as the number of records used in the test and *N_PVC_* is the total number of PVCs which have been annotated.

**Algorithm**	**Database**		**Evaluation**		

	**N**	*N_PVC_*	**Ac(%)**	**Se(%)**	+**P(%)**
Temporal statistics [23]	20	5,677	N/A	91.90	89.46
	44	6,731	N/A	74.51	97.36
PCA [24]	20	5,677	N/A	93.12	94.76
	44	6,731	N/A	92.73	96.92
NNs [18,22,25]	40	6,958	95.20	85.20	92.40
	44	6,958	97.00	93.40	93.30
	7	953	97.04	96.67	97.04
KNN classifier [26]	20	5,677	N/A	97.30	97.70
	44	6,731	N/A	81.60	78.03
Gaussian process [27]	18	2,720	97.10	97.60	97.00
	27	4,080	96.90	84.70	97.50
LHMM	16	6,956	99.20	97.75	96.63
